# Triboelectric Plasma CO_2_ Reduction Reaching a Mechanical Energy Conversion Efficiency of 2.3%

**DOI:** 10.1002/advs.202206125

**Published:** 2022-12-08

**Authors:** Sumin Li, Bao Zhang, Guangqin Gu, Dongyang Fang, Xiaochen Xiang, Wenhe Zhang, Yifei Zhu, Jiao Wang, Junmeng Cuo, Peng Cui, Gang Cheng, Zuliang Du

*Adv. Sci*. **2022**, *9*, 2201633

DOI: 10.1002/advs.202201633

**Figure S4:**
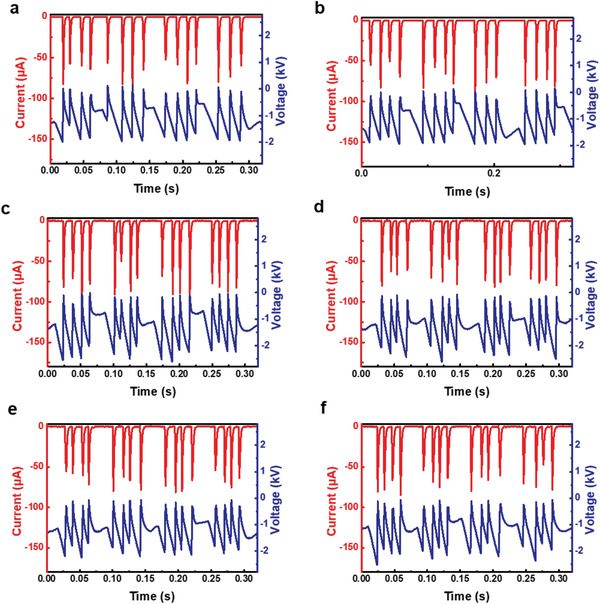
Current and voltage curves of triboelectric plasma at CO_2_ flow rates of 0.2, 1.0, 5.0, 7.5, 10, and 12.5 mL min^−1^. Reaction conditions: TENG rotational speed, 180 rpm; discharge distance, 0.8 mm; room temperature; and atmospheric pressure.

In the original published article, Figure S4f is the same as Figure S4d. Please find the correct Figure S4f below. The authors apologize for any inconvenience caused.

